# A rapid and simple quantitative method for specific detection of smaller coterminal RNA by PCR (DeSCo-PCR): application to the detection of viral subgenomic RNAs

**DOI:** 10.1261/rna.074963.120

**Published:** 2020-07

**Authors:** Pulkit Kanodia, K. Reddisiva Prasanth, Vicky C. Roa-Linares, Shelton S. Bradrick, Mariano A. Garcia-Blanco, W. Allen Miller

**Affiliations:** 1 Interdepartmental Genetics and Genomics, Iowa State University, Ames, Iowa 50011, USA; 2 Plant Pathology and Microbiology Department, Iowa State University, Ames, Iowa 50011, USA; 3 Department of Biochemistry and Molecular Biology, University of Texas Medical Branch, Galveston, Texas 77555, USA; 4 Molecular and Translational Medicine Group, Institute of Medical Research, Faculty of Medicine University of Antioquia, Medellin 050010, Colombia; 5 Programme of Emerging Infectious Diseases, Duke-NUS Medical School, Singapore 169857, Singapore; 6 Institute of Human Infections and Immunity, University of Texas Medical Branch, Galveston, Texas 77555, USA

**Keywords:** truncated RNA, *flavivirus*, *Tombusviridae*, Zika virus, long noncoding RNA

## Abstract

RNAs that are 5′-truncated versions of a longer RNA but share the same 3′ terminus can be generated by alternative promoters in transcription of cellular mRNAs or by replicating RNA viruses. These truncated RNAs cannot be distinguished from the longer RNA by a simple two-primer RT-PCR because primers that anneal to the cDNA from the smaller RNA also anneal to—and amplify—the longer RNA-derived cDNA. Thus, laborious methods, such as northern blot hybridization, are used to distinguish shorter from longer RNAs. For rapid, low-cost, and specific detection of these truncated RNAs, we report detection of smaller coterminal RNA by PCR (DeSCo-PCR). DeSCo-PCR uses a nonextendable blocking primer (BP), which outcompetes a forward primer (FP) for annealing to longer RNA-derived cDNA, while FP outcompetes BP for annealing to shorter RNA-derived cDNA. In the presence of BP, FP, and the reverse primer, only cDNA from the shorter RNA is amplified in a single-tube reaction containing both RNAs. Many positive strand RNA viruses generate 5′-truncated forms of the genomic RNA (gRNA) called subgenomic RNAs (sgRNA), which play key roles in viral gene expression and pathogenicity. We demonstrate that DeSCo-PCR is easily optimized to selectively detect relative quantities of sgRNAs of red clover necrotic mosaic virus from plants and Zika virus from human cells, each infected with viral strains that generate different amounts of sgRNA. This technique should be readily adaptable to other sgRNA-producing viruses, and for quantitative detection of any truncated or alternatively spliced RNA.

## INTRODUCTION

Many positive sense RNA viruses generate 3′ coterminal subgenomic RNAs (sgRNAs) in infected cells. These include many pathogens such as human norovirus, chikungunya, Zika, and dengue viruses, and important plant pathogens such as barley yellow dwarf (BYDV) and maize chlorotic mottle viruses. Most viral sgRNAs, including those of the above viruses, are simply 5′-truncated versions of the viral genome, usually being less than half the length of the full-length genomic RNA (Miller and Koev 2000; Sztuba-Solińska et al. 2011). sgRNAs can serve as mRNAs for translation of open reading frames (ORFs) located downstream from the 5′-proximal ORF(s) that are translated from genomic RNA (Sztuba-Solińska et al. 2011). More recently, sgRNAs have been found that are derived from the 3′ untranslated region (UTR) of the viral genome, and thus function as noncoding sgRNAs (ncsgRNAs) (Iwakawa et al. 2008; Pijlman et al. 2008; Peltier et al. 2012).

For plant viruses in the *Tombusviridae*, *Luteoviridae*, *Solemoviridae*, *Bromoviridae*, *Virgaviridae*, *Benyviridae* families, and the order Tymovirales, and animal viruses in the *Togaviridae* (e.g., chikungunya virus), *Caliciviridae* (e.g., human norovirus), *Astroviridae* (human astrovirus) families, ORFs encoding the RNA-dependent RNA polymerase and associated replicase proteins, located in the 5′ half of the genome, are translated from the viral genomic RNA (gRNA). However, for translation of 5′ distal ORFs that encode proteins required at middle or late stages of infection, such as structural proteins, one or more sgRNAs are generated (Monroe et al. 1993; Koev and Miller 2000; Miller and Koev 2000; Sztuba-Solińska et al. 2011; Royall and Locker 2016; Contigiani and Diaz 2017). For example, the nonstructural polyprotein ORF (including the replicase) of members of *Togaviridae* is translated from gRNA, while the polyprotein ORF encoding structural proteins is translated from a sgRNA that is 3′ coterminal with the gRNA (Strauss and Strauss 1994).

Certain viruses in the *Luteoviridae* (Shen and Miller 2004; Shen et al. 2006; Miller et al. 2015), *Tombusviridae* (Scheets 2000; Iwakawa et al. 2008) and *Benyviridae* (Peltier et al. 2012; Flobinus et al. 2016, 2018) families, and all viruses in the *Flavivirus* genus (Pijlman et al. 2008; Roby et al. 2014) generate ncsgRNAs from the 3′ UTR that play an important role in regulating virus gene expression, virus movement and transmission, with major effects on pathogenicity and symptom development. However, their mechanisms of action are only just beginning to be understood. For example, (i) BYDV sgRNA2 regulates translation of gRNA and sgRNA1 (Shen et al. 2006; Miller et al. 2015), (ii) beet necrotic yellow vein virus sgRNA3 is required for long-distance movement in plants (Peltier et al. 2012), and (iii) subgenomic flavivirus RNAs (sfRNA) interfere with the innate immune systems of mammalian and insect hosts (Schnettler et al. 2012; Bidet and Garcia-Blanco 2014; Bidet et al. 2014; Roby et al. 2014; Manokaran et al. 2015; Donald et al. 2016; Miller et al. 2016; Finol and Ooi 2019).

**FIGURE 1.**
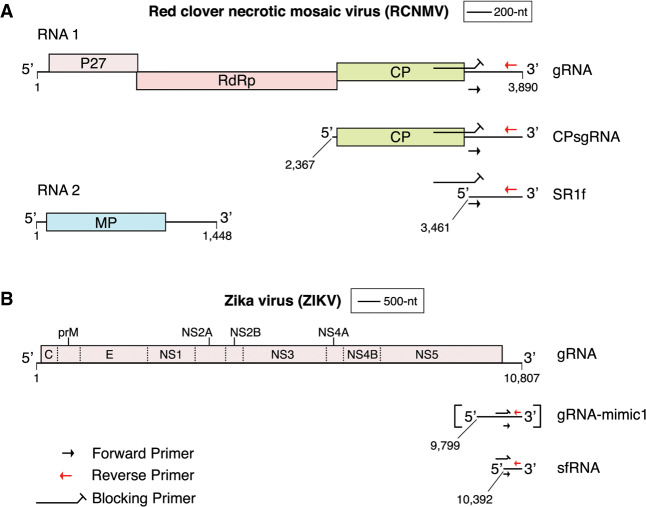
Genome organization of (*A*) Red clover necrotic mosaic virus (RCNMV) and (*B*) Zika virus (ZIKV). ZIKV gRNA-mimic1 (square brackets) was transcribed in vitro and is not produced during ZIKV infection. Approximate positions of DeSCo-PCR primers (not drawn to scale) are shown to depict the approximate location of the primers discussed in Figure 2.

In this study, we detected sgRNAs of red clover necrotic mosaic virus (RCNMV) and Zika virus (ZIKV). RCNMV (Family: *Tombusviridae*, Genus: *Dianthovirus*, Fig. 1A) is a bipartite plant virus with positive-sense single-stranded gRNA1 and gRNA2 (Gould et al. 1981; Hiruki 1987). During infection, a coding sgRNA generated from the 3′ end of gRNA1 serves as the mRNA for viral coat protein translation (Sit et al. 1998). RCNMV also generates a ncsgRNA, SR1f, as a stable degradation product formed by incomplete degradation of gRNA and coat protein sgRNA by a plant 5′ to 3′ exonuclease (Iwakawa et al. 2008; Steckelberg et al. 2018). SR1f is not required for infection of the highly susceptible host plant, *Nicotiana benthamiana*, as an RCNMV mutant that is unable to generate SR1f accumulates substantial levels of the viral genomic RNAs and the coat protein sgRNA (Iwakawa et al. 2008). However, this mutant is unable to accumulate substantially in *Arabidopsis thaliana*.

ZIKV (Family: *Flaviviridae*; Genus: *Flavivirus*; Fig. 1B) usually causes an acute, mild febrile illness, but in the 2015 South and Central American epidemic was found to cause neurological disorders such as microcephaly in infants born to infected mothers and Guillain-Barre syndrome in adults (Beckham et al. 2016; Ferraris et al. 2019). One of the molecular determinants of pathogenicity of ZIKV and other flaviviruses is the sfRNA, which, like SR1f, is an incomplete degradation product of gRNA by a host 5′ to 3′ exonuclease (Pijlman et al. 2008; Silva et al. 2010). RCNMV SR1f and the sfRNAs of ZIKV and other flaviviruses are not required for viral replication but increase virus titer and disease severity (Iwakawa et al. 2008; Pijlman et al. 2008; Moon et al. 2012, 2015; Schnettler et al. 2012; Schuessler et al. 2012; Bidet et al. 2014; Roby et al. 2014; Akiyama et al. 2016; Göertz et al. 2016; Lee et al. 2019). For example, dengue virus disease severity appears to correlate positively with sfRNA level in infected cells. Screening viral mutants that vary in level of sgRNA accumulation is crucial to the understanding of the role of these sgRNAs in viral infection.

In order to (i) decipher the role of ncsgRNA, (ii) identify *cis*- or *trans*-acting RNA elements in a sgRNA, (iii) understand the function of proteins encoded by sgRNAs, (iv) identify promoters required for sgRNA synthesis, (v) undertake field surveys for viral strains with particularly severe symptoms controlled by sgRNA levels, etc., rapid detection of sgRNA and measurement of expression is important. While gRNA can be measured by a simple two-primer based RT-PCR with PCR primers that can hybridize to any region across the gRNA, detection of sgRNAs as distinct from gRNA currently requires more cost- and time-intensive methods, usually northern blot hybridization (Kessler et al. 1990; Amiss and Presnell 2005). In addition, northern blot hybridization is less sensitive compared to RT-PCR and requires several micrograms of total RNA as input. Indirect ways of estimating sgRNA levels include quantitative RT-PCR (qRT-PCR) in which abundance of gRNA, as calculated by gRNA-specific qRT-PCR, is subtracted from total abundance of gRNA and sgRNA, as calculated by qRT-PCR using primers that anneal to their coterminal region (Bidet et al. 2014), or deep sequencing (e.g., Illumina) of total RNA in an infected cell and simply comparing the number of reads that map to the sgRNA region vs the upstream gRNA. However, this too is expensive, time-consuming and requires much bioinformatics analysis post-sequencing. Also, Illumina read counts can vary significantly across a viral genome in the absence of sgRNA (Xu et al. 2019).

To overcome the difficulties and costs associated with the above methods, an RT-PCR approach would be preferable. However, as mentioned above, a simple two-primer based RT-PCR cannot distinguish sgRNA-derived cDNA (sgRNA cDNA) from gRNA-derived cDNA (gRNA cDNA). For an RT-PCR reaction with coterminal RNAs, any primer-pair designed to amplify the sgRNA cDNA will also anneal to the gRNA cDNA, owing to their coterminal ends, resulting in amplification from both, making RT-PCR futile for specific detection of sgRNA. To prevent amplification from gRNA cDNA and enable selective amplification from sgRNA cDNA, we have developed a three-primer based RT-PCR approach, which we name DeSCo-PCR (detection of smaller coterminal RNA by PCR). This method is easy to optimize, relatively simple, quick and inexpensive for specific detection of sgRNAs.

## RESULTS

### Overview of the DeSCo-PCR method

**FIGURE 2.**
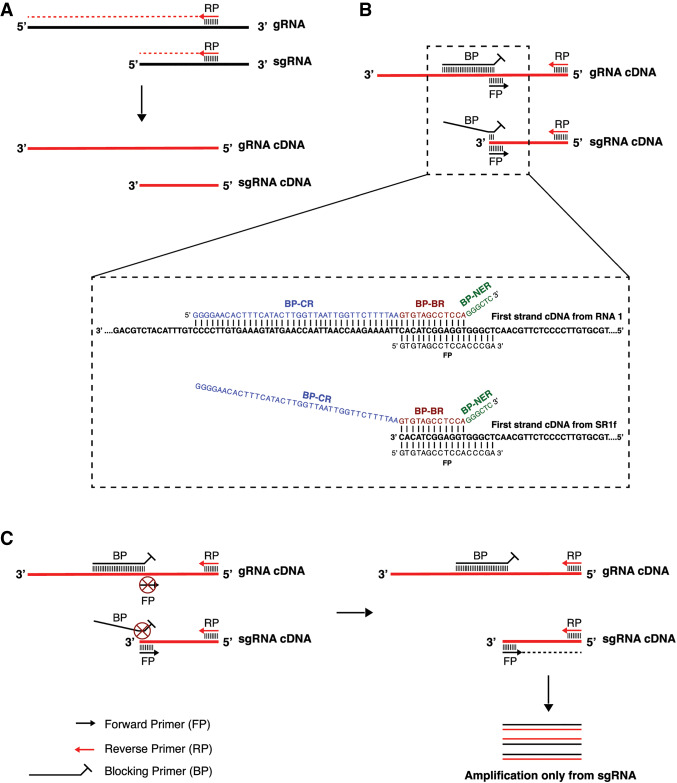
Schematic diagram of DeSCo-PCR. (*A*) First-strand cDNA synthesis (red line) using template-specific reverse primer (RP) annealed to viral positive-strand RNA (bold black line). (*B*) Primer schematics indicating annealing of BP mostly upstream but extending downstream from the 5′ end of sgRNA sequence and annealing of FP to a longer tract starting exactly at the 5′ end of sgRNA sequence. This allows BP to win the annealing competition for gRNA and FP to win the annealing competition for the 5′ end of sgRNA. The dashed box shows the sequences of BP and FP primers and the partial cDNA sequences of RCNMV RNA1 and SR1f to which the primers anneal. (*C*) Primer competition at annealing step and subsequent extension step of DeSCo-PCR. *Vertical* lines represent base-pairing between the primers and the cDNA template. Circled X indicates primer that does not anneal in the presence of competing primer. (gRNA) genomic RNA, (sgRNA) subgenomic RNA, (FP) forward primer, (BP) blocking primer, (CR) competitive region (blue letters), (BR) blocking region (red letters), (NER) nonextendable region (green letters).

DeSCo-PCR utilizes a nonextendable blocking primer (BP) with two amplification primers to prevent amplification of gRNA under conditions that permit amplification of the sgRNA (Fig. 2). Firstly, cDNA to be used as template for DeSCo-PCR is prepared from total RNA, using a virus sequence-specific reverse primer (Fig. 2A). DeSCo-PCR uses three primers (Fig. 2B): (i) a reverse primer (RP) that anneals to gRNA cDNA and sgRNA cDNA at their coterminal 5′ end (complementary to the 3′-coterminal ends of the viral RNAs), (ii) a forward primer (FP), containing the sequence of the 5′ end of the sgRNA, that can anneal to both gRNA cDNA and sgRNA cDNA, and (iii) a long (∼50-nt) forward nonextendable BP containing a contiguous gRNA sequence upstream and downstream from the sgRNA 5′ end followed by a tract of nonviral bases at its 3′ end, which makes it nonextendable by the polymerase (explained in detail below). Under PCR conditions, BP out-competes FP for annealing to gRNA cDNA because it has more bases that can anneal to gRNA cDNA. However, BP is nonextendable and hence, amplification cannot occur from gRNA cDNA. For annealing to sgRNA cDNA, FP outcompetes BP because FP has more bases that can anneal to sgRNA cDNA, resulting in amplification of sgRNA cDNA. Thus, in the presence of all three primers, only sgRNA cDNA is amplified but not the gRNA cDNA (Fig. 2C).

### Blocking primer design for DeSCo-PCR

Blocking primer (BP) is a DeSCo-PCR specific primer that is 50–60-nt long and has three regions (Fig. 2B; dashed box): (i) competitive region (CR), the first ∼40-nt of the primer that can anneal only to gRNA sequence (just upstream of the 5′ end of sgRNA sequence) but not to sgRNA sequence; (ii) blocking region (BR), the ∼10-nt middle region of the primer that can anneal to both gRNA and sgRNA sequences at the 5′ end of the sgRNA (entire sequence of BR is present in the FP); (iii) nonextendable region (NER), the 3′-terminal ∼6-nt of the primer with any nontemplate bases that ensure that the 3′ end of the primer cannot anneal to either the gRNA or sgRNA sequence. Because the 3′ end of the primer cannot anneal, the polymerase cannot extend and hence, amplify from the template. FP and CR-BR sequences of BP can anneal to gRNA sequence. The melting temperature (T_m_) of CR-BR should be significantly higher than FP so that BP will out-compete FP for annealing to gRNA sequence during PCR. FP and BR can anneal to sgRNA sequence. T_m_ of FP should be higher than that of BR so that FP will out-compete BP for annealing to sgRNA sequence. The NER should not be included for any T_m_ calculations. It is preferable to calculate T_m_ according to buffer conditions of the PCR reaction. For example, if Promega GoTaq master mix is used, T_m_ should be calculated using the “T_m_ for Oligos” tool on its website (https://www.promega.com/resources/tools/biomath/) with the appropriate master mix specified.

### General guidelines for optimizing DeSCo-PCR

For optimizing DeSCo-PCR conditions, either in vitro transcribed gRNA and sgRNA can be reverse transcribed and the resulting first-strand cDNA product can be used as template for PCR, or one can use DNA templates with (i) sequence of sgRNA and (ii) at least partial gRNA sequence that includes sgRNA sequence and ∼100 nt upstream of sgRNA. All DeSCo-PCR reactions should be conducted with low ramp-rate for the annealing step of PCR.

The main determinant of PCR parameters is the template concentration. Therefore, in vitro transcribed (IVT) viral RNA concentration or the dilution of cDNA that gives similar band intensity by RT-PCR to that from infected tissues should be determined to serve as a positive control. Next, a gradient PCR with ∼25 cycles should be performed with FP plus RP to determine the maximum annealing temperature (T_m_) that results in amplification from gRNA cDNA (or sgRNA cDNA). At this T_m_, DeSCo-PCR should be carried out with an increasing molar ratio of BP to FP to determine the ratio at which there is amplification predominantly from sgRNA cDNA but not (or only faintly) from gRNA cDNA. A positive control with FP plus RP, and a negative control with BP plus RP should be used with both gRNA cDNA and sgRNA cDNA templates to ensure that any lack of amplification is not because of a failed PCR reaction and any successful amplification is not from BP, respectively. Next, T_m_ can be finely tuned if required, with the selected BP:FP ratio at which sgRNA cDNA is amplified but amplification from gRNA cDNA is completely blocked. Finally, DeSCo-PCR should be conducted with twofold dilution of sgRNA to determine the lower level of detection of sgRNA and the T_m_ can be further fine-tuned accordingly.

To use DeSCo-PCR as a quantitative assay for measuring the relative expression of sgRNA, twofold serial dilutions of sgRNA cDNA can be used as templates for simple- and DeSCo-PCR with varying number of PCR cycles to determine the optimal number of cycles at which DeSCo-PCR reflects the expected sgRNA cDNA dilution.

### Proof-of-concept using in vitro transcribed (IVT) gRNA and sgRNA

To test the concept of DeSCo-PCR, 0.5 pmol each of in vitro transcribed (IVT) RCNMV RNA1, RCNMV SR1f, ZIKV gRNA-mimic1 and ZIKV sfRNA1 were reverse transcribed using either RCNMV reverse primer (RRP) for the RCNMV RNAs or ZIKV reverse primer (ZRP) for the ZIKV RNAs. ZIKV gRNA-mimic1 (Fig. 1B) is a 5′-truncated version of the genomic RNA consisting of the 3′-terminal 1009 nt, to serve as a convenient stand-in for full-length ZIKV RNA for initial RT-PCR experiments. cDNA reaction products were diluted fivefold and 2 µL of these diluted cDNA reaction products were used as template for PCR.

**FIGURE 3.**
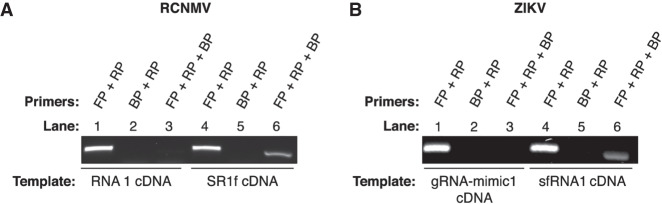
Proof of concept of DeSCo-PCR. (*A*) Selective amplification of cDNA from in vitro transcribed RCNMV SR1f by DeSCo-PCR. (*B*) Selective amplification of cDNA from in vitro transcribed ZIKV sfRNA1 by DeSCo-PCR. (FP) forward primer, (RP) reverse primer, (BP) blocking primer.

Simple PCR with RRP plus RCNMV forward primer (RFP) as a positive control amplified both the cDNA from RNA1 (RNA1 cDNA) and cDNA from SR1f (SR1f cDNA), demonstrating successful amplification under PCR conditions (Fig. 3A, lanes 1,4). PCR with RRP plus RCNMV blocking primer (RBP) did not amplify from either RNA1 cDNA or SR1f cDNA, demonstrating that that RBP is nonextendable under these PCR conditions (Fig. 3A, lanes 2,5). DeSCo-PCR with RRP plus RFP plus RBP resulted in amplification only from SR1f cDNA (Fig. 3A, lane 6) but not from RNA1 cDNA (Fig. 3A, lane 3). Similar results were obtained using the ZIKV primers for DeSCo-PCR on the ZIKV RNA templates (Fig. 3B).

It is noteworthy that an unexpected, very low molecular weight band appeared in the PCR reactions containing BP (Supplemental Fig. S1). To determine whether it is BP-derived primer-dimer, or if it is a nonspecific amplification product, we conducted PCR with BP plus RP, and FP plus BP plus RP, using sfRNA1 cDNA or water as template. The low molecular weight product appeared, even in the absence of a template, indicating that it is a BP-derived “primer-dimer” (Supplemental Fig. S2, lanes 2,3,5,6). In spite of the presence of primer-dimer, detection of sgRNA and measurement of its relative abundance (below) was not affected.

Additionally, there is a small but reproducible increase in mobility of the DeSCo-PCR product compared to the FP-RP PCR product even though both products result from amplification by the FP-RP primer pair (Fig. 3). We found that this difference was due to the presence of the abundant primer-dimer formed only in DeSCo-PCR. We showed this by conducting PCR using ZIKV sfRNA1 cDNA as template with FP plus RP that yields only the band of interest and PCR with BP plus RP that yields only the primer-dimer, mixed these PCR products, and then loaded this mixture in a single well for agarose gel electrophoresis. Mobility of the band of interest from the FP-RP PCR, in the presence of the BP + RP primer-dimer, was identical to that from DeSCo-PCR (Supplemental Fig. S2, lanes 1–4). The reason for the slight mobility change due to the primer-dimer is unclear, but it does not affect the utility of DeSCo-PCR.

### Quantitative analysis for measuring relative amounts of sgRNA by DeSCo-PCR

**FIGURE 4.**
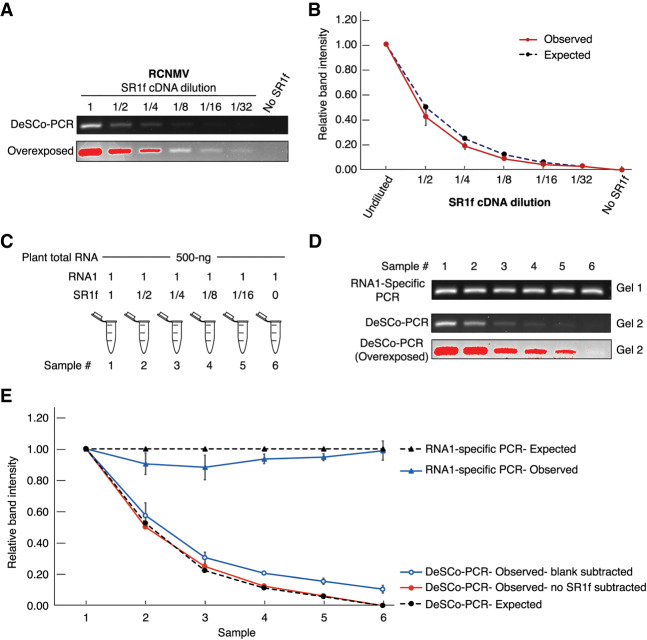
Measurement of relative amounts of in vitro transcribed RCNMV SR1f by DeSCo-PCR. (*A*) DeSCo-PCR gel image. (*B*) Graph of relative amounts of RCNMV SR1f as estimated by relative intensity measurements when only SR1f RNA was used for cDNA synthesis. 1, 1/2, 1/4, 1/8, 1/16, 1/32 denotes the SR1f cDNA dilution, starting with 0.1 pmol of SR1f. (*C*) Composition of RNA mix for reverse transcription containing dilutions of SR1f in the presence of fixed amounts of RCNMV RNA1 and total plant RNA. 1, 1/2, 1/4, 1/8, 1/16 denotes the SR1f dilution. (*D*) DeSCo-PCR gel image of dilutions in *C*. (*E*) Graph of relative RNA abundance as calculated by measuring the band intensities of the PCR products. Gel1: RNA1-specific PCR (both primers upstream of SR1f sequence), Gel2: DeSCo-PCR. Red bands in the gel images denote saturated pixels from overexposing the gel.

To test if DeSCo-PCR can be used as a quantitative assay for measuring relative amounts of sgRNA, we first tested whether PCR of sgRNA-derived cDNA (in the absence of full-length viral cDNA) was quantitative in the presence of the three primers. In vitro transcribed RCNMV SR1f and ZIKV sfRNA1 were reverse transcribed using RRP and ZRP, respectively, and twofold serial dilutions of the resulting cDNA were used as template for PCR. Relative amounts of sgRNA-derived cDNA in each sample was estimated by measuring the relative intensity of each band with respect to that of undiluted sample. DeSCo-PCR with RRP plus RFP plus RBP showed reduction in band intensity with SR1f cDNA dilution (Fig. 4A). Furthermore, relative band intensity, as measured by DeSCo-PCR, precisely reflected the expected SR1f cDNA dilution (Fig. 4B).

We next tested whether DeSCo-PCR can be used as a quantitative assay in the presence of plant total RNA and RCNMV RNA1. Twofold dilutions of IVT SR1f were mixed with a constant amount of *N. benthamiana* total RNA and IVT RCNMV RNA 1 (hence, gRNA and sgRNA are in different ratios). Five hundred nanograms of *N. benthamiana* total RNA was mixed with 0.1 pmol IVT RNA1 and twofold serial dilutions of IVT SR1f starting with an undiluted amount of 0.1 pmol (Fig. 4C). Subsequently, the RNA mixes were reverse transcribed with RRP followed by PCR. RNA1-specific PCR with RNA1-specific forward primer (RCNMV_909_FP) plus RNA1-specific reverse primer (RCNMV_1262_RP) (both far upstream of the sgRNA region of the genome) showed that the band intensity across all samples was uniform, as expected (Fig. 4D, Gel 1). DeSCo-PCR with RRP plus RFP plus RBP that amplifies only SR1f showed reduction in band intensity with SR1f dilution (Fig. 4D, Gel 2). Relative band intensities were used as proxy for measuring the relative amounts of RNA1 or SR1f. The relative amount of RNA1 was mostly uniform across all samples as measured by RNA1-specific PCR, as expected (Fig. 4E). Relative amounts of SR1f (blank subtracted) from DeSCo-PCR reflected the expected SR1f dilutions (Fig. 4E). Relative band intensity calculation by blank-subtracted values shows that there is either a very small amount of amplification that occurs from RNA1 or it is just the background fluorescence. If relative intensities were calculated using no-SR1f sample subtracted values, the estimation of relative amounts of SR1f became even more accurate (Fig. 4E).

**FIGURE 5.**
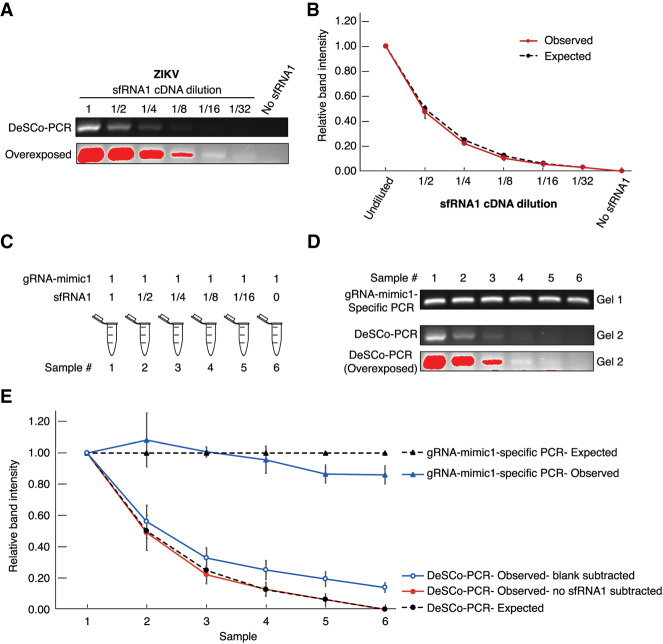
Measurement of relative amounts of in vitro transcribed ZIKV sfRNA1 by DeSCo-PCR. (*A*) DeSCo-PCR gel image. (*B*) Graph of relative amounts of ZIKV sfRNA1 as estimated by relative intensity measurements when only sfRNA1 was used for cDNA synthesis. 1, 1/2, 1/4, 1/8, 1/16, 1/32 denotes the sfRNA1 cDNA dilution, starting with 0.1 pmol of sfRNA. (*C*) Composition of RNA mix for reverse transcription containing dilutions of sfRNA1 in the presence of fixed amounts of gRNA-mimic1. 1, 1/2, 1/4, 1/8, 1/16 denotes the sfRNA1 dilution. (*D*) DeSCo-PCR gel image of dilutions in *C*. (*E*) Graph of relative RNA abundance as calculated by measuring the band intensities of PCR products. Gel1: gRNA-mimic1-specific PCR (both primers upstream of sfRNA1 sequence), Gel2: DeSCo-PCR. Red bands in the gel images denote saturated pixels from overexposing the gel.

We also tested whether the detection of ZIKV sfRNA1 by DeSCo-PCR was quantitative. As for RCNMV, DeSCo-PCR of dilutions of ZIKV sfRNA1-derived cDNA with ZRP plus ZFP plus ZBP showed a reduction in band intensity proportional to the cDNA dilution (Fig. 5A,B). These results show that DeSCo-PCR can precisely measure relative amounts of sgRNA cDNA.

To test if DeSCo-PCR can be used as a quantitative assay in the presence of ZIKV gRNA, twofold dilutions of IVT sfRNA1, starting at 0.1 pmol, were mixed with constant levels (0.1 pmol) of IVT gRNA-mimic1 (Fig. 5C). This RNA mix was reverse transcribed with ZRP followed by PCR. gRNA-mimic1-specific PCR with ZIKV gRNA-specific forward primer (ZIKV_ 9827_FP) plus ZIKV gRNA-specific reverse primer (ZIKV_10115_RP) showed that the band intensity across all samples was uniform, as we observed with RCNMV (Fig. 5D, Gel 1). DeSCo-PCR with ZRP plus ZFP plus ZBP that amplifies only sfRNA1 showed reduction in band intensity with sfRNA1 dilution (Fig. 5D, Gel 2). The relative amount of gRNA-mimic1 was uniform across all samples as measured by gRNA-mimic1-specific PCR, as expected (Fig. 5E). The relative amount of sfRNA1 (blank subtracted) from DeSCo-PCR reflected the expected sfRNA1 dilutions (Fig. 5E). If relative intensities were calculated using no-sfRNA1 sample subtracted values, the estimation of relative amounts of sfRNA1 became even more accurate. Collectively, these experiments show that DeSCo-PCR can quantitatively detect sgRNAs, even in the presence of gRNA, and allow calculation of relative differences in sgRNA/gRNA ratio.

### Specific detection of sgRNA in virus-infected tissues

**FIGURE 6.**
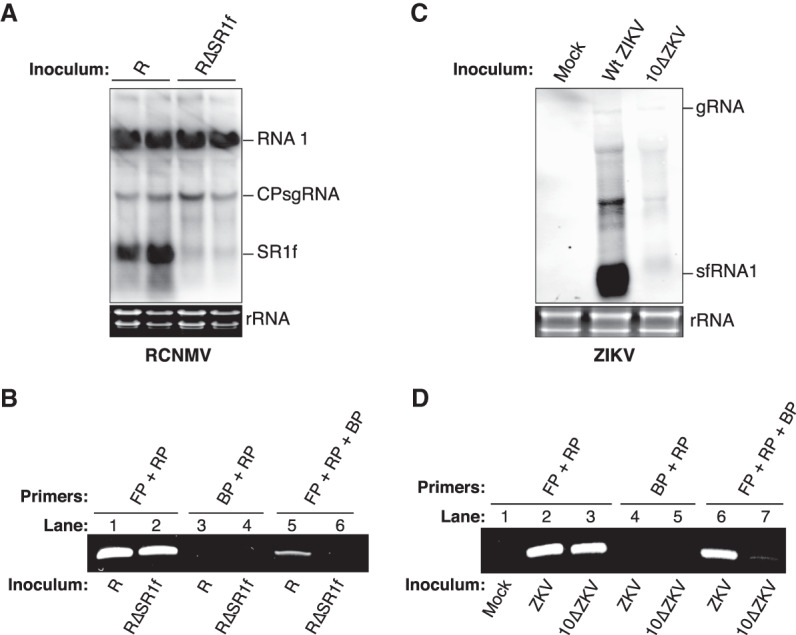
Detection of sgRNAs in virus-infected plants or HeLa cells. (*A*) Northern blot hybridization of total RNA from *N. benthamiana* leaves 14 d after inoculation with wild-type (R) or mutant (RΔSR1f) RCNMV. Stained gel shows ribosomal RNA as loading control for each lane. Duplicate samples are shown for each treatment. (*B*) Detection of SR1f in total RNA from plants infected with indicated wild-type or mutant RCNMV by DeSCo-PCR. Primer combinations used to generate the PCR products are shown *above* each lane of the gel. (*C*) Northern blot hybridization of total RNA from HeLa cells 48 h after inoculation with wild-type (Wt) or mutant 10ΔZIKV. Stained gel shows ribosomal RNA as loading control for each lane. (*D*) Detection of sfRNA1 in total RNA from cells infected with indicated wild-type or mutant ZIKV by DeSCo-PCR. Primer combinations used to generate the PCR products are shown *above* each lane of the gel. (FP) forward primer, (RP) reverse primer, (BP) blocking primer.

We next tested whether DeSCo-PCR could distinguish viral genomic from subgenomic RNA in infected tissues. We first tested RCNMV (R) in the plant host *N. benthamiana*, taking advantage of a viral mutant (RΔSR1f) we constructed, which contains a six-base substitution in its xrRNA structure at the 5′ end of the SR1f sequence, preventing it from generating the noncoding subgenomic SR1f RNA (Iwakawa et al. 2008). Northern blot hybridizations with a probe complementary to the 3′ end of RCNMV RNA1 revealed ample amounts of SR1f from *N. benthamiana* plants infected with wild-type RCNMV, and no (or vanishingly small amounts of) SR1f in plants infected with RCNMVΔSR1f, while both sets of plants accumulated substantial amounts of RCNMV genomic RNA1 and CPsgRNA (Fig. 6A). cDNA was prepared from 1 µg of total RNA from RCNMV-infected and RCNMVΔSR1f-infected *N. benthamiana* leaves using RRP followed by PCR. Because RCNMVΔSR1f has a six-base substitution at the 5′ end of the SR1f sequence, forward and BPs incorporating this substitution, RFP-m1 and RBP-m1, respectively, were used for PCR with cDNA from RCNMVΔSR1f-infected samples. PCR with RRP plus RFP, and RRP plus RFP-m1 resulted in amplification from both RCNMV-infected and RCNMVΔSR1f-infected cDNA samples, respectively, confirming successful virus infection (Fig. 6B, L1 and L2). PCR with RRP plus RBP, and RRP plus RBP-m1 primers did not result in amplification showing that the RBP and RBP-m1 are nonextendable under PCR conditions (Fig. 6B, L3 and L4). DeSCo-PCR with RRP plus RFP plus RBP amplified only from RCNMV-infected cDNA samples (Fig. 6B, L5) while DeSCo-PCR with RRP plus RFP-m1 plus RBP-m1 resulted in no amplification from RCNMVΔSR1f-infected cDNA samples (Fig. 6B, L6) demonstrating that SR1f is detected only in wild-type RCNMV-infected plants and not in RCNMVΔSR1f-infected plants.

We next tested ZIKV RNA accumulation in HeLa cells, taking advantage of a mutant, 10ΔZIKV (deletion of nts 10,650 to 10,659 in the 3′UTR) that produces a lower ratio of sfRNA1/gRNA than wild-type ZIKV (Shan et al. 2017b). Northern blot hybridization with a 3′ probe complementary to ZIKV RNA revealed much greater levels of sfRNA1 in cells infected with wild-type virus than with the mutant. In this case, the genomic RNA levels were also reduced in 10ΔZIKV infection, but the sfRNA1 was virtually undetectable by northern blot hybridization in 10ΔZIKV-infected cells (Fig. 6C). cDNA was prepared from 1 µg total RNA from mock-infected, wild-type ZIKV-infected and 10ΔZIKV-infected HeLa cells using ZRP. PCR of the resulting cDNA template with ZRP plus ZFP primers amplified both ZIKV-infected and 10ΔZIKV-infected cDNA samples, but not from mock-infected cDNA samples, as expected (Fig. 6D, lanes 1–3). There was no amplification using ZRP plus ZBP primer pairs, confirming that the ZBP is nonextendable under the PCR conditions (Fig. 6D, lanes 4,5). DeSCo-PCR with ZRP plus ZFP plus ZBP primers yielded a product from cDNA from cells infected with wild-type ZIKV (Fig. 6D, lane 6), but only a very faint band from 10ΔZIKV-infected cells (Fig. 6D, lane 7), reflecting the ratios of sfRNA1/gRNA observed by northern blot hybridization and published previously (Shan et al. 2017b). Collectively, these experiments demonstrate that DeSCo-PCR can be used for specific, quantitative detection of sgRNAs from hosts in different kingdoms infected by unrelated viruses.

## DISCUSSION

DeSCo-PCR is a simple, quick, inexpensive and sensitive assay that can selectively amplify a viral sgRNA from a pool of RNA containing host total RNA, viral gRNA and other sgRNAs. Even though northern blot hybridization has certain advantages (e.g., the entire sequence of sgRNA need not be known and it can detect gRNA and multiple sgRNAs simultaneously), DeSCo-PCR can easily detect sgRNAs in a variety of experimental settings to rapidly screen for sgRNA production. Similar to northern blot hybridization, DeSCo-PCR can be used for measuring relative abundance of sgRNAs in different experimental conditions such as those from mutant viral genomes or in transgenic hosts. While it does not measure absolute amounts of RNA, DeSCo-PCR quantitatively measures relative amounts of sgRNA and can detect differences in ratios of sgRNA:gRNA between different virus isolates. The advantages of DeSCo-PCR are particularly beneficial for experiments where several viral mutants or isolates need to be screened rapidly to identify the relative amount of a particular sgRNA each viral isolate produces. For examples, 10ΔZIKV produces sfRNA1 at a very low sfRNA1/genomic RNA ratio and has reduced accumulation and attenuated pathogenicity compared to wild-type ZIKV. This makes 10ΔZIKV a vaccine candidate against ZIKV infection (Shan et al. 2017a,b).

In addition, DeSCo-PCR can be used by clinics or laboratories that do not have access to radioisotopes, expensive nonradioactive chemiluminescent northern blot reagents or an imager required for detection of fluorescent probes used in northern blots. For viruses that require replication to generate sgRNAs, DeSCo-PCR could be used as a quick or confirmatory assay to determine whether a virus is replicating, without need for measuring increases in total RNA or infectious units over time.

DeSCo-PCR is not limited to virology. It can be used for detecting smaller coterminal RNAs of any origin. Coterminal RNAs are present in eukaryotes as truncated RNA isoforms transcribed by alternative transcription start sites (TSS) or may be produced as alternatively spliced RNA isoforms. These truncated mRNA isoforms may differ in their 5′ UTR, affecting their stability and translation efficiency or differ in their encoded protein domains, affecting their localization, function and protein stability (Rojas-Duran and Gilbert 2012; Wang et al. 2016; Galipon et al. 2017). For example in humans, adenosine deaminases acting on RNA (ADARs) are involved in RNA editing, and the ADAR1 gene produces two coterminal mRNA isoforms, ADAR1-p150 and ADAR1-p110 from an interferon-inducible promoter and a constitutive promoter, respectively (Galipon et al. 2017). Additionally, next-generation sequencing and computational analysis are often used to identify, predict functions and determine differential expression of these transcript isoforms with a certain degree of confidence (Kandoi and Dickerson 2017, 2019; Qin et al. 2018). However, these analyses are often followed by molecular assays for validation and DeSCo-PCR provides a simple alternative to northern blot hybridization for confirming the production of a truncated RNA isoform with coterminal ends and measuring their relative abundance.

Because DeSCo-PCR involves competition between blocking and forward primers for selective annealing to gRNA or sgRNA cDNA, it may be possible to design primers that tolerate a few mismatched bases at the 5′ end of sgRNA in cases where the exact 5′ end nucleotide of the sgRNA has not been determined precisely. Also, it may be possible to use a BP terminating in a dideoxynucleotide (Sanger et al. 1977) to make it universally nonextendable, instead of the mismatched 3′ terminal sequence on our BPs. This may eliminate the production of BP-derived primer-dimer and therefore, make DeSCo-PCR adaptable to qRT-PCR. In addition, a BP with a few locked nucleic acid (LNA) (Koshkin et al. 1998; Ballantyne et al. 2008; Veedu et al. 2008) nucleotides in the BP-CR region would increase its binding affinity to gRNA cDNA, helping BP to out-compete FP for annealing to gRNA cDNA at lower annealing temperatures, which would be more optimal for amplification. However, use of dideoxynucleotides or LNAs would increase primer costs many-fold. Although presently DeSCo-PCR cannot be used for absolute quantification of the number of copies of a sgRNA by qRT-PCR because of amplification of primer-dimer (Supplemental Fig. S1), it can reliably be used to quantitatively compare the relative abundance of sgRNAs of different virus strains or mutants in a highly sensitive manner. Similar to northern blot hybridization, DeSCo-PCR requires some optimization with every virus, but this can be done in a short time (2 or 3 d) (Table 1). In summary, DeSCo-PCR provides a simple, readily optimized, cost-effective method for rapid, sensitive quantification of viral subgenomic RNAs in only a limited amount of total RNA and without the use of expensive and hazardous chemicals.

**TABLE 1.**
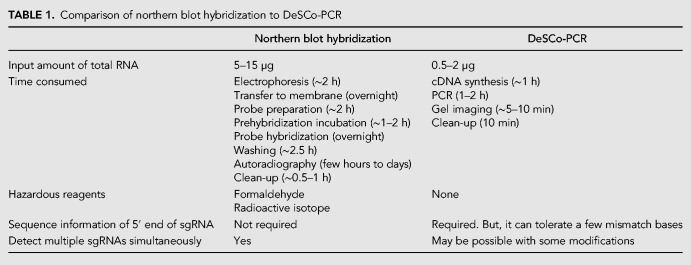
Comparison of northern blot hybridization to DeSCo-PCR

## MATERIALS AND METHODS

### Oligonucleotide synthesis

All primers were synthesized by Integrated DNA Technologies and purified by standard desalting. Sequences and genomic positions of primers that were used for construction of pRC169c, pRSR1f, pR1m1, ZIKV gRNA-mimic1 PCR product, and ZIKV sfRNA1 PCR product are listed in Supplemental Table S1. Sequences and genomic positions of primers that were used for all RT-PCR experiments, including DeSCo-PCR, are listed in Supplemental Table S2.

### Plasmid construction

Full-length infectious cDNA clones of RCNMV Australian strain RNA1 (pRC169) and RNA2 (pRC2|G) (Xiong and Lommel 1991; Sit et al. 1998) were kindly provided by Dr. Tim L. Sit and Dr. S.A. Lommel. pRC169 and pRC2|G are cDNA clones with a T7-promoter for in vitro transcription of infectious RNA1 and RNA2, respectively. pRC169 was sequenced by Sanger sequencing and was found to contain several base changes compared to the sequence from NCBI (GenBank: J04357). Two of the base changes, at positions 3462 and 3494, were present near the 5′ end of SR1f and therefore, were changed from C to T and G to A, respectively, using Q5 Site-Directed Mutagenesis kit (NEB #E0554) according to manufacturer's protocol with primers 3UTR_R1_corrected_for and 3UTR_R1_corrected_rev. The corrected plasmid, pRC169c, was used as template for construction of pRSR1f and pR1m1, and as template for in vitro transcription of infectious RCNMV RNA1.

#### pRSR1f

pRSR1f is a cDNA clone with T7-promoter followed by SR1f sequence for in vitro transcription of SR1f. A Q5 Site-Directed Mutagenesis kit (NEB #E0554) was used according to manufacturer's protocol. A DNA fragment with the T7 promoter sequence, vector sequence and SR1f sequence was amplified from pRC169c with the following PCR reaction composition and conditions: Q5-hot start high fidelity 2× master mix (1×), T7-rev primer (0.5 µM), SR1f_for primer (0.5 µM), pRC169c as template (10 ng); initial denaturation at 98°C for 30 sec; 25 cycles of denaturation at 98°C for 10 sec, annealing at 60°C for 30 sec, extension at 72°C for 2.5 min; final extension at 72°C for 2 min. This was followed by ligation, according to manufacturer's protocol, to circularize the PCR product. Subsequently, the plasmid was transformed in *E. coli* sigma 10 cells and colonies were screened in LB-agar plates with ampicillin. Plasmids were extracted from selected colonies and the sequence was verified by Sanger sequencing.

#### pR1m1

pR1m1 is an infectious cDNA clone of RCNMV RNA1 (RNA1-m1) that does not generate SR1f during infection. pR1m1 has a six-base substitution (“TGTAGC” to “ACGTTG”) in pRC169c (nts 3462 to 3467) that disrupts the xrRNA structure required for SR1f production (Iwakawa et al. 2008). A Q5 Site-Directed Mutagenesis kit (NEB #E0554) was used according to manufacturer's protocol. The DNA fragment was amplified by PCR with the following reaction composition and conditions: Q5-hot start high fidelity 2× master mix (1×), SR1f.m1_for primer (0.5 µM), SR1f.m1_rev primer (0.5 µM), pRC169c as template (10 ng); initial denaturation at 98°C for 30 sec; 25 cycles of denaturation at 98°C for 10 sec, annealing at 59°C for 30 sec, extension at 72°C for 4 min; final extension at 72°C for 2 min. This was followed by ligation, according to manufacturer's protocol, to circularize the PCR product. Subsequently, the plasmid was transformed in *E. coli* sigma 10 cells and colonies were screened in LB-agar plates with ampicillin. Plasmids were extracted from selected colonies and the sequence was verified by Sanger sequencing.

All RCNMV cDNA clones were linearized at a unique SmaI restriction site at the precise 3′ end of the RCNMV 3′ UTR prior to in vitro transcription.

#### ZIKV gRNA-mimic1 PCR product

ZIKV gRNA-mimic1 PCR product is a DNA fragment with a T7 promoter followed by a partial sequence of ZIKV gRNA (nts 9799 to 10807). It was amplified from pFLZIKV (Shan et al. 2016) using the primers NS5 (+) forward primer 1 and sfRNA (−) reverse primer. ZIKV gRNA-mimic1 PCR product was used for in vitro transcription to make noninfectious ZIKV gRNA-mimic1 that was used for DeSCo-PCR experiments.

#### ZIKV sfRNA1 PCR product

ZIKV sfRNA1 PCR product is a DNA fragment with a T7-promoter followed by the sequence of ZIKV sfRNA1 (nts 10392 to 10807). It was amplified from pFLZIKV (Shan et al. 2016) using the primers sfRNA (+) forward primer and sfRNA (−) reverse primer. ZIKV sfRNA1 PCR product was used for in vitro transcription to make ZIKV sfRNA1 that was used for DeSCo-PCR experiments.

### In vitro transcription

One µg linearized plasmid for all RCNMV constructs, 200 ng ZIKV sfRNA1 PCR product, and 500 ng ZIKV gRNA mimic1 PCR product were used as templates for in vitro transcription using MEGAscript T7 Transcription kit (Invitrogen #AM1334) followed by DNase treatment according to manufacturer's protocol. The transcription reaction was carried out at 37°C for 4 h and DNase treatment at 37°C for 30 min. Subsequently, RNA was purified using Zymo RNA Clean & Concentrator -5 kit (Zymo Research #R1015) and eluted in nuclease-free water.

### Virus inoculation and RNA extraction

#### RCNMV

*Nicotiana benthamiana* plants at the four-leaf stage were used for inoculations. Two leaves per plant were inoculated. Per leaf, 1 µg in vitro-transcribed (IVT) RCNMV RNA1 plus 1 µg IVT RCNMV RNA2 were mixed in 10 mM sodium phosphate buffer (pH 6.8) and rubbed on the leaves. These are referred to as RCNMV-infected plants that make SR1f. Similarly, 1 µg IVT RCNMV RNA1-m1 plus 1 µg IVT RCNMV RNA2 were mixed in 10 mM sodium phosphate buffer (pH 6.8) and rubbed on the leaves. These are referred to as RCNMVΔSR1f-infected plants that do not make SR1f. For Figure 6, leaves from RCNMV- and RCNMVΔSR1f-infected *N. benthamiana* were collected at 5 d post inoculation (dpi) for PCR and at 14 dpi for northern blot hybridization, pulverized and total RNA was extracted using Zymo Direct-zol RNA Miniprep (Zymo Research #R2051).

#### ZIKV

Hela cells were seeded at a density of 3 × 10^5^ cells per well in a six-well plate. One day later, cells were infected with the wild-type (ZIKV-Cambodia) or mutant (10ΔZIKV) virus at an MOI of 3. After 48 h post-infection, cells were washed with PBS and total RNA was extracted from cells using the Direct-zol RNA MiniPrep kit (Zymo Research).

### cDNA synthesis

Amount of in vitro transcribed RNA or plant/cell total RNA that were reverse transcribed is indicated in the results section. RNA (IVT or total RNA) and virus-specific reverse primer (15 pmol; same reverse primer as used for DeSCo-PCR) were mixed in nuclease-free water to 12 µL and incubated at 65°C for 5 min, transferred to ice followed by addition of 4 µL reaction buffer, 1 µL RiboLock, 2 µL 10 mM dNTPs and 1 µL RT enzyme from RevertAid First Strand cDNA Synthesis kit (Thermo Scientific #K1621). The reaction mix was incubated at 42°C for 60 min followed by enzyme deactivation at 70°C for 5 min. The cDNA reaction products from IVT RNAs were diluted fivefold and considered as “undiluted samples” for experiments with serially diluted templates while cDNA reaction products from total RNA from infected samples were not diluted but used as is for PCR.

### PCR

GoTaq G2 green master mix (Promega #M7823) was used for all PCR reactions. Simple PCR with RP plus FP as positive control, RP plus BP as negative control, DeSCo-PCR with RP plus FP plus BP, and gRNA-specific PCR were carried out in a thermocycler with the capability of controlling the ramp rate. Ramp rate of 0.5°C per second was used for the PCR reactions specified below. A 20 µL PCR reaction mix was prepared with 2-µL template and final concentration of each of the primers, if used, were as follows: 0.2 µM RP, 0.2 µM FP, 4 µM BP. BP: FP = 20: 1 was determined, empirically, as optimum for RCNMV and ZIKV, for successful DeSCo-PCR to selectively amplify sgRNA cDNA and completely block amplification from gRNA cDNA (data not shown). The primers used are mentioned below and the primer sequences can be found in Supplemental Table S2. PCR conditions were as follows:

#### RCNMV

98°C (2 min); 18 cycles of 98°C (30 sec), 65°C (20 sec, ramp rate = 0.5°C/sec), 72°C (30 sec); 72°C (2 min); 4°C hold. Primers used were RFP, RRP, RBP, RFP-m1, and RBP-m1.

#### RCNMV (RNA1-specific PCR)

98°C (2 min); 18 cycles of 98°C (30 sec), 60°C (20 sec), 72°C (30 sec); 72°C (2 min); 4°C hold. Primers used were RCNMV_909_FP and RCNMV_1262_RP.

#### ZIKV (with IVT templates)

98°C (2 min); 22 cycles of 98°C (30 sec), 66.5°C (20 sec, ramp rate = 0.5°C/sec), 72°C (40 sec); 72°C (2 min); 4°C hold. Primers used were ZFP, ZRP, and ZBP. ZIKV gRNA-specific PCR was carried out in the same conditions as above with primers ZKV_9827_FP and ZKV_10115_RP.

#### ZIKV (with infected samples as templates)

98°C (2 min); 30 cycles of 98°C (30 sec), 65°C (20 sec, ramp rate = 0.5°C), 72°C (40 sec); 72°C (2 min); 4°C hold. Primers used were ZFP, ZRP, and ZBP.

PCR reaction products were run on a 1% agarose gel, with SYBR Safe DNA gel stain (Invitrogen #S33102), in 1× TBE buffer and visualized on a Bio-Rad Gel doc. The gel images shown were cropped to show the band of interest. One thing to note is that all DeSCo-PCR performed with RCNMV and ZIKV resulted in amplification of BP-derived primer-dimer but it did not affect the relative band intensity measurement.

### Measurement of relative expression of sgRNA

When the agarose gels were imaged for quantitative analysis, the exposure time was set for maximum duration at which no saturating intensity was observed in the amplified bands. Fiji software (ImageJ) was used to measure band intensity. Intensity of the background was measured from three separate regions of the gel where no band/DNA is expected, and the values were averaged. The averaged background intensity, referred to as “blank,” was subtracted from the intensity of each band from gRNA-specific PCR. For DeSCo-PCR, either the “blank” values or the band intensity of “No sgRNA” samples were considered as background intensity and were subtracted from the band intensity of each sample. This was done three times for each gel and background-subtracted values from the three measurements were averaged. The final values were normalized with respect to the band intensity of undiluted cDNA. The values obtained from DeSCo-PCR are the relative band intensity representing the relative amount of sgRNA in each sample. For all results shown with the relative measurement of sgRNA, PCR was carried out three times and the values for the relative band intensities were averaged and plotted on a graph using Microsoft Excel. Error bars represent the standard deviation of the relative band intensities obtained from three PCR reactions.

### Radiolabeled RNA probe preparation

A DNA template with SP6-promoter for transcribing a radiolabeled RNA probe that can hybridize to a positive sense strand of RCNMV RNA1 3′ UTR (nts 3605 to 3800) was prepared by PCR with the following composition and conditions: An amount of 50 µL reaction with GoTaq G2 green master mix (1×), R1.3UTR.for (0.2 µM, 5′-TCG GAC CCT GGG AAA CAG GT-3′), R1.3UTR.SP6.rev (0.2 µM, 5′-GATATTTAGGTGACACTATAGAGGTATGCGCCCTCTGAGC-3′), pRC169 as template (10 ng); initial denaturation at 95°C for 2 min; 25 cycles of denaturation at 95°C for 30 sec, annealing at 56°C for 30 sec, extension at 72°C for 30 sec; final extension at 72°C for 5 min. The underlined bases in the primer sequence represent SP6 promoter sequence. The amplified product was purified using QIAquick PCR Purification kit (Qiagen #28104) and used as a template for making a radiolabeled probe using MEGAscript SP6 Transcription kit (Invitrogen #AM1330) with the following reaction composition: An amount of 2 µL 10× reaction buffer, 2 µL 5mM AUG mix, 2.5 µL 0.1 mM CTP, 50 ng DNA template, 0.5 µL RNase OUT (Invitrogen #10777019), 2 µL SP6 enzyme, 2.5 µL CTP (ɑ-^32^P; PerkinElmer #BLU008X250UC). The reaction was incubated at 37°C for 3 h followed by DNase treatment with 1 µL Turbo DNase at 37°C for 15 min and a radiolabeled RNA probe was purified using Micro Bio-spin 30 columns (Bio-Rad #732-6251) and stored at −20°C.

### Northern blot hybridization

#### RCNMV

An amount of 9.5 µg total RNA from noninoculated leaves of RCNMV- and RCNMVΔSR1f-infected *N. benthamiana* were mixed with an equal volume of 2× RNA loading dye (NEB #B0363S), denatured by incubating at 70°C for 10 min and 5 min on ice and loaded on a 1.2% agarose-formaldehyde gel (1.2% [w/v] agarose, 20 mM sodium phosphate buffer [pH 6.8], 8 mL of 37% formaldehyde per 100 mL of gel). Electrophoresis was carried out at 100 V for 2 h in running buffer (74 mL of 37% formaldehyde per 1 L of running buffer, 20 mM sodium phosphate buffer [pH 6.8]). Integrity and equal loading of RNA were verified by visualizing the gel on a Bio-Rad Gel doc. The gel was washed in sterile water for 5 min at room temperature (RT) and blotted to a nitrocellulose membrane by the capillary transfer method overnight using 10x saline-sodium citrate (SSC) buffer (Invitrogen #AM9763). Post-transfer, the membrane was washed in 5× SSC for 5 min at RT, dried on a paper towel, and UV-crosslinked in StrataGene UV Stratalinker 1800 using the “Auto Cross Link” option. The membrane was placed in a glass cylindrical bottle and incubated in 5 mL hybridization buffer (50% [v/v] formamide, 5× SSC buffer, 0.2 mg/mL polyanetholsulphonic acid, 0.1% [w/v] SDS, 20 mM sodium phosphate buffer [pH 6.8]) at 65°C for 1 h in a hybridization oven (VWR). The buffer was discarded, and fresh 5 mL hybridization buffer was added to the bottle with 5 µL radiolabeled RNA probe. Probe hybridization was carried out overnight in a hybridization oven at 65°C. Post hybridization washes were carried out in a hybridization oven as follows: two washes with 50 mL high salt concentration buffer (1× SSC, 0.1% [w/v] SDS) at RT for 20 min, two washes with 50 mL low salt concentration buffer (0.2× SSC, 0.1% [w/v] SDS) at 68°C for 20 min, and one wash with 50 mL 0.1× SSC buffer at RT for 20 min. The membrane was dried on a paper towel, covered in a saran wrap and placed inside the phosphor cassette with phosphor screen, imaged by autoradiography using Bio-Rad PharosFX Plus Molecular Imager.

#### ZIKV

An amount of 5 μg of total RNA from mock and ZIKV-infected cells was mixed with 2× formaldehyde loading buffer (Thermo Fisher Scientific), and denatured by incubating at 65°C for 15 min and 2 min on ice. Electrophoresis was performed in 1% denaturing agarose gel and stained with ethidium bromide. After electrophoresis, the gel was incubated in the alkaline buffer (0.01 N NaOH, 3 M NaCl) for 20 min and subsequently transferred to a Biodyne B nylon membrane (Thermo Fisher Scientific) by upward transfer. The membrane was crosslinked using a UV Stratalinker and blocked at 42°C using ULTRAhyb Oligo hybridization for 1 h while rotating. Blots were probed overnight rotating at 42°C with a Biotin-labeled DNA probe prepared as described in Soto-Acosta et al. (2018). After hybridization, the membrane was washed in wash buffer for 15 min at 42°C four times. The blot was incubated for 1 h at room temperature with IRDYE 800CW streptavidin (LI-COR Biosciences) in Odyssey Blocking Buffer (LI-COR Biosciences) with 1% of SDS. Later the membrane was washed three times with TBS buffer containing 0.1% tween, and the membrane was scanned using an LI-COR Odyssey.

## SUPPLEMENTAL MATERIAL

Supplemental material is available for this article.

## Supplementary Material

Supplemental Material
