# Supplementary material for: A rapid and simple quantitative method for specific detection of smaller coterminal RNA by PCR (DeSCo-PCR): application to the detection of viral subgenomic RNAs
Source: RNA. 2020 Jul;26(7):888–901. doi: 10.1261/rna.074963.120 (PMC7297113; doi:10.1261/rna.074963.120)
Supplement: Supplemental Material [file supp_26_7_888__index.html]

A rapid and simple quantitative method for specific Detection of Smaller Co-terminal RNA by PCR (DeSCo-PCR): Application to the detection of viral subgenomic RNAs — A rapid and simple quantitative method for specific detection of smaller coterminal RNA by PCR (DeSCo-PCR): application to the detection of viral subgenomic RNAs — Supplemental Material 

# A rapid and simple quantitative method for specific detection of smaller coterminal RNA by PCR (DeSCo-PCR): application to the detection of viral subgenomic RNAs

## Supplemental Material

- Supplemental\_Data.docx
