# Supplementary material for: A rapid and simple quantitative method for specific detection of smaller coterminal RNA by PCR (DeSCo-PCR): application to the detection of viral subgenomic RNAs
Source: RNA. 2020 Jul;26(7):888–901. doi: 10.1261/rna.074963.120 (PMC7297113; doi:10.1261/rna.074963.120)
Supplement: Supplemental Material [file supp_074963.120_Supplemental_Data.docx]

**SUPPLEMENTARY DATA**

**Supplementary Tables**

**Supplementary Table S1: Primers used for construction of DNA templates for *in vitro* transcription.**

| **Primer** | **Nucleotide position ^a^** | **Sequence (5’----3’) ^b, c^** |
| --- | --- | --- |
| 3UTR_R1_corrected_for | 3487- 3506 | GGGGAAC**A**CGCAGTCTCGCC |
| 3UTR_R1_corrected_rev | 3448- 3486 | TCTTGCAACTCGGGTGGAGGCTAC**A**CTTAAAAGAACCAA |
| T7-rev | - | CTATAGTGAGTCGTATTAGGGTACCGAGC |
| SR1f_for | 3461-3480 | GTGTAGCCTCCACCCGAGTT |
| SR1f.m1_for | 3465-3485 | **TTG**CTCCACCCGAGTTGCAAG |
| SR1f.m1_rev | 3429-3464 | **CGT**CTTAAAAGAACCAATTAACCAAGTATGAAAGTG |
| NS5 (+) forward primer 1 | 9799-9819 | GCTAATACGACTCACTATAGGGCTCCCACCACTTCAACAAGC |
| sfRNA (-) reverse primer | 10787-10807 | AGACCCATGGATTTCCCCACA |
| sfRNA (+) forward primer | 10392-10416 | GCTAATACGACTCACTATAGTGTTGTCAGGCCTGCTAGTCAGCC |
| ^a^ nucleotide position is with respect to the gRNA sequence of RCNMV (GenBank J04357) and ZIKV (Genbank KU955593).  ^b^ Sequence that was substituted in the template is in bold.  ^c^ T7-promoter sequence is underlined | | |

**Supplementary Table S2: Primers used for RT-PCR and DeSCo-PCR.**

| **Primer** | **Nucleotide position ^a^** | **Sequence (5’----3’) ^b^** |
| --- | --- | --- |
| RCNMV reverse primer (RRP) | 3871- 3890 | GGGGTACCTAGCCGTTATAC |
| RCNMV forward primer (RFP) | 3461- 3477 | GTGTAGCCTCCACCCGA |
| RFP-m1 | 3461- 3477 | GACGTTGCTCCACCCGA |
| RCNMV blocking primer (RBP) | 3423- 3478 | GGGGAACACTTTCATACTTGGTTAATTGGTTCTTTTAAGTGTAGCCTCCAgggctc |
| RBP-m1 | 3423- 3478 | GGGGAACACTTTCATACTTGGTTAATTGGTTCTTTTAAGACGTTGCTCCAgggctc |
| RCNMV_909_FP | 909- 926 | AAGCGGGCCAGTAGAGTC |
| RCNMV_1262_RP | 1244-1262 | TCTCCATTGCACAGGTTTC |
| ZIKV reverse primer (ZRP) | 10691-10711 | GCGTCAATATGCTGTTTTGCG |
| ZIKV forward primer (ZFP) | 10392-10410 | GTGTTGTCAGGCCTGCTAG |
| ZIKV blocking primer (ZBP) | 10356-10410 | GGGTCCACACCTGGAGTGCTATAAGCACCAATCTTAGTGTTGTCAGGCCacgatc |
| ZIKV_9827_FP | 9827-9846 | CAAGGACGGGAGGTCCATTG |
| ZIKV_10115_RP | 10095-10115 | GTTCCACACCACAAGCATGTC |
| ^a^ nucleotide position is with respect to the gRNA sequence of RCNMV (GenBank J04357) and ZIKV (Genbank KU955593).  ^b^ bases in small case letters in blocking primers do not anneal to either gRNA or sgRNA making the primer non-extendable. | | |

**Supplementary Figures**

**
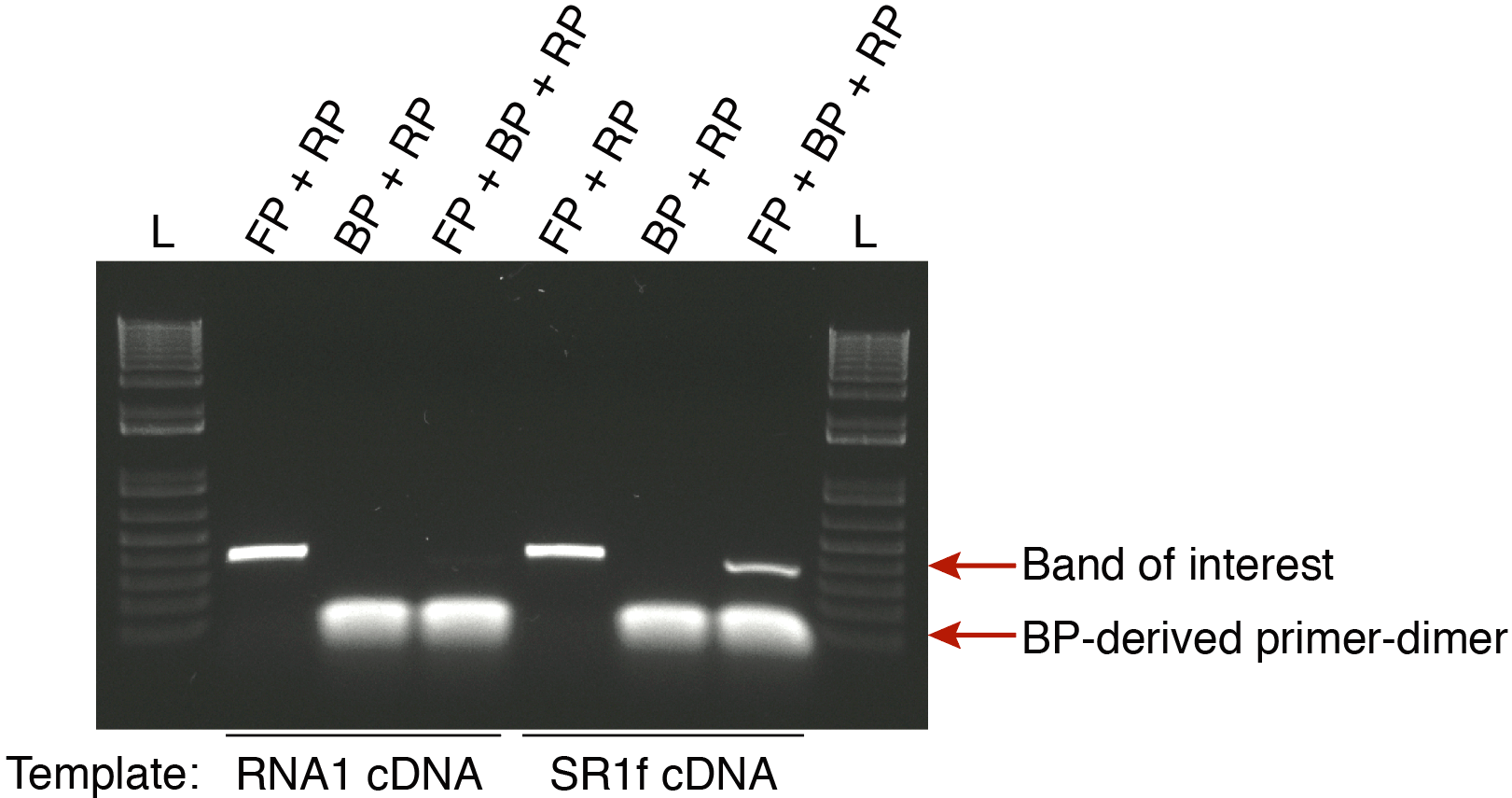
Supplementary Figure S1:**  Uncropped DeSCo-PCR gel image from Figure 3A shows amplification of primer-dimer from PCR in samples with blocking primer. RCNMV RNA1-derived cDNA and SR1f-derived cDNA were used as templates for the PCR reaction. FP: forward primer, BP: blocking primer, RP: reverse primer, L: Invitrogen 1kb plus DNA ladder.

**
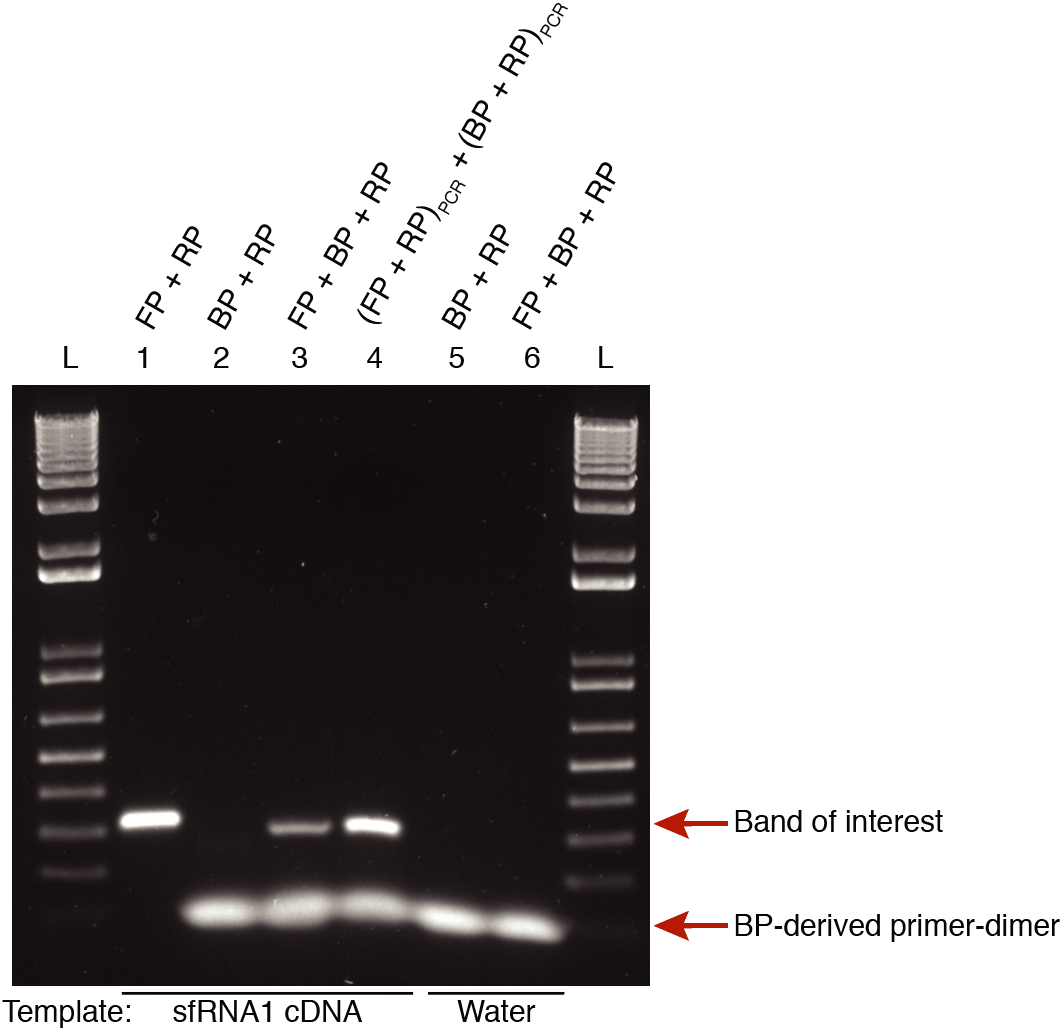
Supplementary Figure S2:**  Presence of a BP-derived “primer-dimer” in sample slightly increases the mobility of the band of interest. PCR using BP plus RP yields a low molecular weight band in the presence of either ZIKV sfRNA1-derived cDNA, or no template (water) (L2-3, L5-6). In addition, the band of interest migrates slightly faster in the presence of the primer-dimer in DeSCo-PCR reactions (L3) compared to the same sized FP-RP PCR product (L1). This is shown by mixing the FP-RP PCR product that yields only the band of interest (L1) with BP-RP PCR product that yields only the primer-dimer (L2) and loading the mixture in a single well for agarose gel electrophoresis (L4). Mobility of the band of interest from the FP-RP PCR, in the presence of primer-dimer (L4), was similar to that from DeSCo-PCR (L3) and faster than that of FP-RP alone (L1). FP: forward primer, BP: blocking primer, RP: reverse primer, L: Invitrogen 1kb plus DNA ladder.
